# F237. DOPAMINERGIC EFFECTS ON HIERARCHICAL PREDICTION ERRORS AND CONNECTIVITY DURING SOCIAL LEARNING

**DOI:** 10.1093/schbul/sby017.768

**Published:** 2018-04-01

**Authors:** Daniel Hauke, Andreea Diaconescu, Katharina Wellstein, Sara Tomiello, Lionel Rigoux, Jakob Heinzle, Klaas Enno Stephan

**Affiliations:** 1 University of Basel, UPK; 2 University of Zurich, ETH Zurich; 3 Max Planck Institute for Metabolism Research

## Abstract

**Background:**

Persecutory delusions (PD) constitute core symptoms in psychosis that may emerge from aberrant learning and inference about others’ intentions. Computational assays that use generative models of electrophysiological data to probe this learning process and its underlying neuronal mechanisms, in particular the effects of dopamine (DA) on synaptic plasticity, could provide mechanistic insights into the emergence of PD in psychosis. More importantly, they could enable prediction of individual treatment response to DA antagonists and thus help to address an important problem of clinical management of psychosis.

**Methods:**

We tested 137 healthy volunteers (mean age: 22 ± 3) in a double-blind, placebo-controlled, between subject pharmacological study: placebo (n = 47), DA precursor L-Dopa (n = 45), and DA receptor antagonist amisulpride (n = 45). Electroencephalography was recorded using a 128-channel Brain-Vision system.

Participants performed a social learning task that required learning about an adviser’s intentions and how they changed over time. Subsequently, we modeled participants’ behavior with the hierarchical Gaussian filter (HGF), a model in which learning is driven by hierarchical prediction error (PE) updates: At the first level, positive PEs indicate that advice was better than expected (advice PE: aPE). At the second level, a positive PE signals that the adviser’s intentions were less stable than predicted (volatility PE: vPE). Using the trial-wise estimates from the HGF, we performed single-trial EEG analyses of PE activity at sensor and source levels. We also examined DA effects on effective connectivity with dynamical causal modelling (DCM). To this end, we divided event-related potentials (ERPs) according to PE magnitudes into 2 bins corresponding to positive and negative PEs.

**Results:**

At the sensor-level, we identified distinct temporal profiles of hierarchical PEs (peak effects: aPE at 112ms, vPE at 276ms). In source space, three sources showed significant effects for both PEs: anterior temporo-parietal junction (TPJ), dorsal middle cingulate cortex (MCC) and supplementary motor cortex (SMA).

To identify the connections that convey PEs, we compared two DCM families that allowed input to different nodes of the network, and different modulatory effects of PE magnitude. The family with input entering the SMA and propagating via MCC to TPJ explained aPE-evoked activity best, whereas the family with input into the TPJ and propagating in the opposite direction best described the effects of vPE-evoked activity. Bayesian model selection identified the winning model for aPE effects; this model proposed PE magnitude modulations of input gain and effective connectivity from TPJ to MCC, and MCC to SMA. Conversely, a model with connectivity modulation from MCC to TPJ best described the effects of vPE. Second, we investigated the impact of dopaminergic perturbations of the network by comparing DCM parameters of the winning models across pharmacological groups. Post hoc t-tests revealed that DA impacted on aPE-induced perturbations only, which is in line with previous findings that aPEs are represented in dopaminergic regions while vPEs are likely encoded by activity in cholinergic regions. Specifically, DA modulated TPJ-MCC and SMA-MCC connectivity.

**Discussion:**

Model-based analysis of EEG data in a social learning task detects DA effects on connectivity, even when behavior (accuracy, reaction time) was not affected by the drugs. Currently, we are extending our computational approach to first-episode schizophrenia patients, where we hope to use parameters from neuronal and behavioral models to predict individual treatment response.

